# The predictive role of psychological toughness and adaptability on the actual well-being of mothers with handicapped children

**DOI:** 10.4314/ahs.v22i1.71

**Published:** 2022-03

**Authors:** Mir Hamid Salehian

**Affiliations:** Department of Physical Education, Tabriz branch, Islamic Azad University, Tabriz, Iran

**Keywords:** Psychological toughness, adaptability, actual well-being, handicapped children

## Abstract

**Background and Aim:**

Handicapped children cause psychological problems for mothers. As mothers' actual well-being is so important in the family and society, the aim of this research was to predict the role of psychological toughness and adaptability on the actual well-being of mothers with handicapped children.

**Materials and Methods:**

The research was descriptive correlational study with volunteer mothers of handicapped children (n=150). Data collection tools were: Ahwaz Kiamarsi et al. (1998) psychological toughness questionnaire, Connor and Davidson adaptability scale (2003), Lyubomerisky and Leper actual well-being scale (1999) and Diener life satisfaction scale (2009). Pearson correlation coefficient and multiple linear regression analysis were used to analyze the data using SPSS 21 version.

**Results:**

The results showed that psychological toughness and its components (commitment, control, struggle) have a significant positive relationship with the actual well-being of mothers with handicapped children and its dimensions (actual well-being and life satisfaction) and is able to predict their well-being.

**Discussion:**

Therefore, it can be said that by promoting adaptability, mothers with handicapped children can resist and overcome stressors as well as factors that cause many psychological problems. By reducing psycho-emotional problems in mothers, mental well-being and life satisfaction are improved.

**Conclusion:**

The actual well-being of mothers with handicapped children can be predicted by their psychological toughness and adaptability.

## Introduction

Physical disability is one of the major disabilities of children in many countries^1^. The World Health Organization uses disability as a general term for injuries, functional and participatory limitations that lead to dysfunction of the body and deprives the individual of participation in daily personal and social activities^2^. Awareness of any problems, developmental delays, or differences in the child can be very hard on parents. Therefore, parents experience intense emotions when they realize their child's disability. Meanwhile, mothers often experience more stress than fathers because of their special role in the birth and care of a disabled child. Fathers usually do not express their feelings as much as mothers, while mothers show a wide range of emotions such as anger, sadness, crying and grief^3^. Studies have shown that mothers with disabled children have lower levels of general health and lower well-being, lower quality of life and psychological capital, lower life satisfaction and higher levels of stress compared to mothers of healthy children, and have lower psychological well-being^3^–^8^.

According to statistics released by the World Health Organization, there are 6.5 million people with disabilities living in the world, of which about 70% are in developing countries. In Iranian society, according to the Welfare Organization, about 7% of the population suffers from some kind of disability and annually about 25,000 children with disabilities are born in the country with severe physical and motor disorders^9^.

Active well-being is a broad concept that includes experiencing pleasant emotions, low levels of negative mood, and high life satisfaction. In short, actual well-being has two components, emotional and cognitive. Emotional or emotional components include the balance of positive emotion (well-being) and negative emotion. The cognitive component (life satisfaction) is also the cognitive assessment that each person makes of the overall quality of life or its specific areas.^10^ Therefore, people with actual well-being have a sense of life satisfaction, high positive emotion and low negative emotion^10^,^11^. Adaptability is one of the factors that help a person to cope with difficult and stressful life situations and protect people against obstacles, maintaining optimism and positive emotions in difficulties, and avoiding erosive strategies pathological disorders and life difficulties^12^. Connor and Davidson^13^ describe adaptability as the ability to balance bio-psychological balance in dangerous situations. They see adaptability not only as adaptability to threatening conditions, but also as the active and effective participation of the individual in the environment. In addition, researchers believe that adaptability is a kind of self-healing with positive emotional and cognitive consequences, which in itself plays an important role in adaptation and satisfaction with life as much as possible^14^.

In general, adaptability includes: maintaining calm when faced with pressure, being resilient to the unwanted events. In addition, resilient people are normally able to think creatively and flexibly about problem solving, and seek help from others when needed and help others in times of crisis. These people have degrees of health and independence. They believe in their ability to change the environment^15^. Cohn et al.^16^ showed that adaptability has an effect on increasing positive emotions, well-being and reducing negative emotions. In another study, Suri et al.^17^ stated that psychological well-being is affected by personality factors, including adaptability, which can predict 27% of psychological well-being. The results of the study of Sattarpour et al.^18^ showed that adaptability has a significant positive relationship with the actual well-being of mothers of students with mental disabilities and its dimensions (actual well-being and life satisfaction) and is able to predict about 11% of them.

Pardel et al.^19^ concluded that psychological adaptability has a significant effect on the feeling of well-being and improving the quality of life of mothers of children with ADHD. Poursardar et al.^20^ showed that adaptability leads to greater life satisfaction by reducing negative emotions and increasing mental health.

It should be noted that another variable that is closely related to actual well-being is psychological toughness. Stubbornness is a combination of beliefs about self and the world using the existential theories of personality, which consists of three components: challenge, control and commitment^21^. In fact, psychological rigidity is a single structure that deals with the integrity of its components and creates homogeneity in them^22^.

Psychological stubbornness is born of the knowledge that a person has more resources to respond to stress. In other words, it is a fundamental sense of control that allows the stubborn person to achieve a number of useful and effective strategies. Also, psychological stubbornness causes a person to consider their stresses in a realistic and far-sighted way. Ultimately, psychological stubbornness reinforces an optimistic view of psychological stress.

In fact, psychological adaptability is a shield against intense physiological arousal due to stressful events.^23^ Psychological toughness has a negative effect on stress and a positive effect on satisfaction. Toughness may act as an important coping force against the effects of pressure for change^24^. Studies show that there is a significant relationship between psychological toughness with physical and mental health, mental well-being, emotion control, anger, anxiety, depression and quality of life^25^–^28^.

Baghchaghi et al.^29^ concluded that psychological toughness is an important predictor of cognitive emotion regulation strategies that lead to improved quality of life in mothers of autistic children. Also, Asghari and Mamizadeh^30^ showed that psychological toughness can predict 51% of psychological well-being. Obviously, all people can achieve well despite the hardships and sufferings that lie ahead. Mothers with handicapped children are no exception.

In view of the above, and considering that the mother has a key role in maintaining the psychological and social balance of the family, and creating tension and pressure in the mother affects other family members, and given that the mother has an important role in mental health of family members It is very important to address the situation of mothers with handicapped children.

Considering the mentioned effects of individual psychological and personality capacities (adaptability and psychological toughness) on the well-being of mothers of exceptional children and the importance of actual well-being for mothers with handicapped children, as well as insufficient research in this field, the predictive role of psychological toughness and adaptability in the actual well-being of mothers with handicapped children was assessed.

## Materials and methods

This study according to the purpose is a basic study, and in terms of data collection and analysis, the method is descriptive and correlational design. The statistical population of this study was all mothers with handicapped children covered by the General Welfare Office of Tabriz in 2020 (400 people according to the statistics of the General Welfare Office of Tabriz). The sample size was estimated to be 196 based on Cochran's formula and the samples were selected by simple random sampling.

### Inclusion criteria

1- Having a handicapped child. 2- Having a minimum diploma. 3- Age range 30 to 45 years; 4- Having an average economic status 5- To have complete satisfaction to participate in the research. And exclusion criteria: There was no completion of the questionnaire or any particular problem during the research.

After receiving permission from the General Welfare Office of Tabriz and coordination with the authorities, the selected mothers (196 participants) were invited to attend the amphitheater of the General Welfare Office of Tabriz at the appointed time. After the presence of mothers with handicapped children, the researcher explained the purpose of the research, the method of completing the questionnaire and after obtaining cooperation and informed consent, three questionnaires were presented to mothers continuously and together to complete all. Questionnaires were designed without a name and were collected after completion.

Following tools have been used to collect information: Ahwaz Toughness Inventory (AHI): This questionnaire was developed by Kiamarsi et al.^23^ and it has 20 items and its purpose is to evaluate the degree of stubbornness and its factors in individuals. The way of scoring this questionnaire is that the options are never, rarely, sometimes and often will get scores of 4, 3, 2, 1, respectively. The sum of the total scores of these questions is considered as the toughness score of the subject and the higher this score is, the higher the toughness of the respondent and vice versa. In this questionnaire, questions 1 to 9 are related to the commitment factor, questions 10 to 16 are related to the control factor and questions 17 to 20 are related to the struggle factor.

Cronbach's alpha coefficient was used to measure the internal consistency of “Ahwaz Toughness Questionnaire” and based on the findings of alpha coefficients for the whole sample, male and female subjects were 0.76, 0.76 and 0.74, respectively.^23^

Adaptability Questionnaire: Connor and Davison Adaptability Scale were used to measure adaptability^13^. This scale consists of 25 items, each of which is graded on a five-point Likert scale from zero to four and has a total score. Validity (by factor analysis and convergent and divergent validity) and reliability (by retesting and Cronbach's alpha) adaptability scale has been achieved by the test manufacturers in different normal and atrisk groups. In Iran, Mohammadi^31^ reported the reliability of this scale as 0.89 and its validity 0.64. In the present study, the reliability of the adaptability scale was calculated to be 0.86 using Cronbach's alpha method.

Lyubomirsky & Lepper Active Well-being Scale^32^ and the Diener Life Satisfaction Scale^33^ are among the most appropriate scales for assessing actual well-being. These two self-assessment tests were used to assess the actual well-being of retired teachers. Therefore, the total score of well-being and life satisfaction will be the level of actual well-being^34^.

The actual well-being scale has 4 items and people respond to it on a five-point scale. This scale measures people's well-being independently and in comparison with their peers. The internal validity of the scale is reported from 0.85 to 0.95 based on Cronbach's alpha^35^,^36^. The Life Satisfaction Scale has five items and is a single factor. This scale is designed to measure the cognitive dimension of actual well-being. The validity of the scale was 0.87 with Cronbach's alpha method and 0.82 with two-month interval with retest method. This scale has been validated in Iran by Bayani et al.^36^ using the Cronbach's alpha method of 0.83 and the retest method of 0.69.

### Data analysis

Data were analyzed using SPSS software and Pearson correlation and multiple regression tests.

## Results

According to the findings of Table 1, the mean actual well-being is 18.49±4.43, psychological toughness is 59.24±5.92 and adaptability is 55.24±8.04

**Table 1:** - Descriptive statistics of variables

Variables	M	Std. dev.	Skew	Elong
General Welfare	18.49	4.43	-0.17	-0.48
Happy Activity	9.10	4.35	-0.14	-0.9
Life Satisfaction	9.85	3.6	0.13	-0.36
Psychological toughness	59.24	5.92	-0.42	0.14
Adaptability	55.24	8.04	-0.37	-0.36

The result of table 2 shows that show that there is a relationship between psychological toughness with actual well-being (p<0.05, r = 0.50), actual well-being (p<0.05, r = 0.31) and life satisfaction (p<0.05, r = 0.80) There is a significant positive relationship. Between adaptability with actual well-being (p <0.05, r = 0.39), actual well-being (p<0.05, r = 0.31) and life satisfaction (p<0.05, r = 0.32) There is a significant positive relationship.

**Table 2:** Pearson correlation test results for variable relationships

Variables	General Welfare	General Welfare	Life Satisfaction	Psychological toughness	Adaptability
General Welfare	r	1			
	p				
Happy Activity	r	0.822	1		
	p	0.001			
Life Satisfaction	r	0.801	0.318	1	
	p	0.001	0.001		
Psychological toughness	r	0.499	0.311	0.493	1
	p	0.001	0.001	0.001	
Adaptability	r	0.391	0.323	0.364	0.317
	p	0.001	0.001	0.001	0.001

The result of table 4 shows that the multiple correlation co-efficient is 0.56 and the determination coefficient is 0.32. In fact, 32% of the variance of actual well-being is explained by the variables of psychological toughness and adaptability. The value of the Watson camera is 1.8. Since this value is between 1.5 and 2.5, it is concluded that the criterion variable is not self-correlated and the errors are independent of each other. Also, the significance level of F test is equal to 0.001. Considering that the level of significance of F-test is less than 0.05, it shows that there is a significant linear relationship between the criterion variable and the predictor variables.

**Table 4:** Table of regression coefficients

	Non-standardized coefficients standardized coefficients			T	Sig.
	B	Std. Dev.	Beta		
Fixed value	-7.723	2.365		-2.171	0.011
Psychological toughnes	0.435	0.047	0.357	6.246	0.001
Adaptability	0.225	0.04	0.309	3.759	0.001

According to table 3, it is concluded that psychological toughness (p = 0.001 and beta = 0.43) and adaptability (p = 0.001 and beta = 0.22) have a positive effect on the actual well-being of mothers with handicapped children. They are meaningful. Thus, psychological toughness and adaptability variables can predict the actual well-being of mothers with handicapped children. Psychological toughness with a standardized coefficient (beta) of 0.35 has the greatest effect on predicting actual well-being.

**Table 3:** Correlation table, camera-Watson and F for the effect of adaptability and psychological toughness on mothers' actual well-being

Multiple correlation coefficient	determination coefficient	Watson camera	F	Sig.
0.562	0.320	1.812	41.541	0.001

## Discussion

The aim of this study was to investigate the predictive role of psychological toughness and adaptability in the actual well-being of mothers with handicapped children. The results showed that psychological toughness and its components (commitment, control, struggle) have a significant positive relationship with the actual well-being of mothers with handicapped children and its dimensions (actual well-being and life satisfaction) and is able to predict their well-being.

There is no report in the research literature about the role of psychological toughness in the actual well-being of mothers with handicapped children; But the result obtained with the results of Florian et al.,^25^ Salehian and Ghadiri,^26^ Azarian et al.,^27^ Shokouhi Fard et al.,^28^ Baghchaghi et al.^29^ There is a significant positive relationship between psychological toughness with physical and mental health, psychological well-being, control of negative emotions, mental well-being and improving quality of life. Families with handicapped children are under more stress than families with healthy children. In addition, the first person who has direct contact with the child is the mother.

While constant maintenance and the need to provide special conditions for growth and exposure to pressures such as, stereotyped behaviors and lack of self-care skills in these children weaken the normal functioning of the mother and also mothers react negatively to their child's behaviors, it causes high levels of stress and marital conflicts, separation, divorce, low self-esteem, decreased well-being and life satisfaction in mothers with handicapped children^7^.

Explaining this research finding, it can be stated that how the mother deals with this issue (birth crisis of a disabled child) depends on the personality traits of the mother. Toughness, defined by Kubasa as a personality construct, is a combination of components of commitment, control, and struggle that contribute to individuals' physical and mental health by coping with traumatic events and modifying life stressors, also, mothers who score high in the struggle component view life problems and stressors as an opportunity for growth and change rather than failure. This attitude to stress and problems effectively deal with it and prevents the weakening of the immune system and vulnerability of people and brings them mental and physical health, and as a result with positive emotions and life satisfaction in mothers with handicapped children.

The results also showed that adaptability has a significant positive relationship with the actual well-being of mothers with handicapped children and its dimensions (actual well-being and life satisfaction) and is able to predict their actual well-being. This finding suggests that by promoting adaptability, mothers can resist and overcome stressors as well as factors that cause many psychological problems.

Adaptability also ensures the psychological well-being of individuals by modulating and mitigating factors such as stress and depression.^37^ The finding is consistent with the results of the research of Sattarpour et al.^18^ showed that explained the inclusion of significant variance in the actual well-being (well-being and practical satisfaction and life satisfaction) of mothers of mentally retarded students.

Also, it is consistent with the results of studies by Cohn et al.,^16^ Souri et al.,^17^ Pardel et al.,^19^ Poursardar et al.,^20^ that adaptability on increasing positive emotions, well-being and decreasing negative emotions, well-being has a greater psychological effect on life and satisfaction. Explaining this research finding, it can be stated that adaptability, which means coping with problems in traumatic events and being flexible in responding to the pressures of daily life, is a trait that varies from person to person and can grow or decrease over time^38^.

Adaptability strengthens successful coping with negative experiences by increasing levels of positive emotions. Based on this, it seems that resilient people look at problems creatively and flexibly, plan to solve them and do not hesitate to ask for help from others when needed, and have complete resources to deal with the problems that these factors cause the person have a life satisfaction^39^. It can be concluded that the intervention is effective and improves the cognitive ability and resilient of blind athletes^40^.

Therefore, it can be said that by promoting adaptability, mothers with handicapped children can resist and overcome stressors as well as factors that cause many psychological problems. By reducing psycho-emotional problems in mothers, mental well-being and life satisfaction in mothers are improved.

The results of simultaneous multiple regression analysis also showed that psychological toughness and adaptability explained 31% of the actual welfare variance of mothers with handicapped children and had significant predictive power and psychological adaptability and adaptability with standardized beta coefficient (0.43 and 0.25) have the most and the least effect on the prediction of actual well-being, respectively. Therefore, the most important predictor variable in this study is psychological toughness.

In fact, it can be said that psychological toughness reduces the level of anxiety and depression by equipping a person with a shield to deal with stressful situations, and by activating problem-based coping strategies in stressful situations, it makes a person look at events with more optimism. As a result, it reduces the risk of diseases and increases pleasant emotions, well-being and positive feelings about life and well-being in the individual, and as a result, increases life satisfaction in mothers.

## Conclusion

The results of the present study showed that psychological toughness and productivity can significantly predict changes in actual well-being in mothers with handicapped children and strong variables in explaining the actual well-being of mothers with handicapped children. Therefore, it is recommended that mothers and their families be taught how to increase them, and that government institutions and organizations provide the necessary support for them. Also, due to the limitations of the research, which is a sample only of mothers with handicapped children, it is suggested that such research be conducted in mothers of other groups with special needs, fathers and in other cities.

## References

[1] Tsai C-F, Guo H-R, Tseng Y-C, Lai D-C (2018). Sex and geographic differences in the prevalence of reported childhood motor disability and their trends in Taiwan. BioMed Research International.

[2] Aurora U (2014). Study for determining laterality in children with motor disabilities in adapted physical activities. Proced-Soc Behav Sci.

[3] Salehi M, Shirin K, Hagh Doost N (2011). The compaire of mental health, coping strategies and well- being between mother with physically handicap and normal children. J Psych Res.

[4] Murphy NA, Christian B, Caplin DA, Young PC (2007). The health of caregivers for children with disabilities: caregiver perspectives. Child: care, health and development.

[5] Bayrami M, Movahedi Y, Kodayari F (2015). The compersion of psychlogical capital and quality of life between mothers of healthy and handicaped children. Iran J Rehab Research in Nurs.

[6] Aghajani MJ, Hosain Khanzadeh A, Akbari B, Mirarzgar Ms (2017). The effect of the Islamic Positive Thinking Training on Anger Management and Life Satisfaction in Mothers of Exceptional children. Middle Eastern Journal of Disability Studies.

[7] Lee J (2013). Maternal stress, well-being, and impaired sleep in mothers of children with developmental disabilities: a literature review. Res Develop Disab.

[8] Khodabakhshi KA (2012). Consoling and psychotherapy in disability rehabilitations (Application of theories).

[9] Nassir Aqa B, Hojjatollah F, Ali Reza Fazili M (2012). Spiritual Intelligence and Subjective Well-Being. Ravanshenasi Va Din.

[10] Pavot W, Diener E (2008). The satisfaction with life scale and the emerging construct of life satisfaction. J Positive Psych.

[11] Connor KM, Davidson JR (2003). Development of a new adaptability scale: the Connor-Davidson Adaptability Scale (CD-RISC). Depress Anxiety.

[12] Luthar SS, Cicchetti D, Becker B (2000). The construct of adaptability: a critical evaluation and guidelines for future work. Child Dev.

[13] Bogar CB, Hulse-Killacky D (2006). Resiliency determinants and resiliency processes among female adult survivors of childhood sexual abuse. J Counsel Develop.

[14] Cohn MA, Fredrickson BL, Brown SL, Mikels JA, Conway AM (2009). Well-being unpacked: positive emotions increase life satisfaction by building adaptability. Emotion.

[15] Hossein S, Elahe H, Javad E (2013). The correlation of resiliency and optimism with psychological well-being. International J Beh Sci.

[16] Sattarpour FI, Jamali YG, Leyla H, Hamed M (2018). Investigating the Role of Predictability of Adaptability, Social Support and Intellectual Aptitude in Subjective Well-Being of Student's Mothers with Intellectual Disability. J Know Health.

[17] Pardel H, Dideh Roshani S, Hejazipour M (2014). The effectiveness of mental adaptability training on well-being and improving the quality of life of mothers of children with ADHD. Third National Conference on Mental Health and Wellness; Quchan (Iran).

[18] Poursardar F, Abbaspour Z, Abdi Zarrin S, Sangari AA (2012). The effect of adaptability on enhancing mental health and life satisfaction. Sci J Yafte.

[19] Soodeh K, Maedeh Sadat E, Mohammad Hossein A, Mahnaz S (2017). The relationship between toughness and adaptability with self-efficacy in crisis managers of Red Crescent society. J Res Relief.

[20] Kobasa SC (1979). Stressful life events, personality, and health: an inquiry into toughness. J Person Soc Psych.

[21] Mirzamohammadi Z, Mohsenzadeh PD F, Arefi PD M (2017). The Relationship between Parenting Styles, Psychological Toughness, and Students' Adaptability. Quart J Family Res.

[22] Rush MC, Schoel WA, Barnard SM (1995). Psychological resiliency in the public sector:” Toughness” and pressure for change. J Voc Behav.

[23] Florian V, Mikulincer M, Taubman O (1995). Does toughness contribute to mental health during a stressful real-life situation? The roles of appraisal and coping. Journal of Personality and Social Psychology.

[24] Salehian M H, Ghadiri S (2019). The Effect of Cognitive Emotion Regulation and Psychological Well-being on Athletic Performance of Professional and Semi-professional Athletes. Sport Psych Stud.

[25] Azarian A, Farokhzadian AA, Habibi E (2016). Relationship between psychological toughness and emotional control index a communicative approach. Intern J Med Res Health Sci.

[26] Shokohifard s, hamid n, soodani m (2017). The Effect of Toughness Training on Quality of Life of Teacher Training Female Students. Know Res Appl Psych.

[27] Baghchaghi F, Vakili S, Kashani Vahid L (2019). A study of the relationship between quality of life and psychological toughness with cognitive emotion regulation strategies of mothers of autistic children. Family Conference, Autism Disorder and Associated Challenges.

[28] Asghari Ebrahimabad M, Mamizade M (2018). An Investigation into the Role of Psychological Flexibility and Toughness in Explaining Soldier's Psychological Well-Being. Res Clinic Psych Counsel.

[29] Mohammadi M, Jazayeri A, Rafie AH, Joukar B, Pourshahbaz A (2006). adaptability factors in ndividuals at risk for Substance abuse. J Psych (Tabriz University).

[30] Lyubomirsky S, Lepper HS (1999). A measure of subjective well-being: Preliminary reliability and construct validation. Soc Indicat Res.

[31] Diener E, Diener E (2009). Assessing subjective well-being: Progress and opportunities. Assessing well-being: The collected works of Ed Diener.

[32] Salehi M, Janbozorgi M, Tabatabaei SKRZ (2016). The Effectiveness of Islamic Hope Therapy on the Subjective Well-being of the People with Multiple Sclerosis Compared with Snyder Hope Therapy. Psychol Relig.

[33] Aghababai N, Farahani HA, Rahiminejad A, Fazeli Mehrabadi A (2009). Spirituality and subjective well-being in university students and clergies. J Psychol Sci.

[34] Bayani AA, Koocheky AM, Goodarzi H (2007). The reliability and validity of the satisfaction with life scale. J Iran Psych.

[35] Salehian MH, Pour Haji S, Harzandi H, Ghanati P (2021). The Effect of Cognitive Emotion Regulation on Athletic Performance of Semi-professional Athletes. The Seconal National Conference on Sport Sciences.

[36] Khalatbari J, Bahari S (2010). Relationship between adaptability and satisfaction of life. J Educ Psych.

[37] Karami J, Sanjabi A, Karimi P (2017). The Prediction of Life Satisfaction among the Elderly Based on Adaptability and Well-being. Ag Psych.

[38] Dana A, Soltani N, Fathizadan A, Rafiee S (2019). Effectiveness of Mindfulness-Based Cognitive Behavioral Therapy on Cognitive and Adaptability Abilities of Blind Athletes. J Sport Psychol Stud.

